# Approaches to vascularizing human brain organoids

**DOI:** 10.1371/journal.pbio.3002141

**Published:** 2023-05-08

**Authors:** Bing Ye

**Affiliations:** 1 Life Sciences Institute, University of Michigan, Ann Arbor, Michigan, United States of America; 2 Department of Cell and Developmental Biology, University of Michigan, Ann Arbor, Michigan, United States of America

## Abstract

The development of brain organoids has been a boon to neurological research, but working out how to add blood vessels to organoids has posed a challenge. This Perspective looks at innovative approaches to brain organoid vascularization, including those put forward in a 2020 PLOS Biology paper.

## Teaser

A major challenge in brain organoid technologies is the lack of vasculature. In recent years, innovative approaches have been taken to meet this challenge. A 2020 paper published in *PLOS Biology* exemplifies the approaches used in this booming field.

This article is part of the *PLOS Biology* 20th Anniversary Collection.

Brain organoids, which are derived from pluripotent stem cells, can show us what occurs in spontaneous organogenesis from stem cells as well as what happens during induction by well-defined cues. As such, they offer an alternative approach to understanding brain development. Moreover, the use of induced pluripotent stem cells (iPSCs) for deriving human brain organoids offers a unique opportunity of modeling brain disorders. iPSC-derived brain organoids can be used to investigate the causality between genetic variants and certain brain phenotypes, to model diseases that are of heterogeneous etiology, and even to develop therapeutic approaches [1,2]. While we are far from modeling the complete developmental process of the brain, impressive steps have been made toward modeling aspects of this process.

A number of challenges limit the utility of brain organoids, among which is the lack of vasculature. When an organoid that lacks vasculature grows, hypoxia, as well as a lack of nutrients and removal of metabolites, becomes increasingly severe in the interior of the organoid, leading to cell stress and death. Consistent with this, cells in vasculature-lacking brain organoids express markers for stress-associated genes, indicating increased levels of cell stress. Thus, the absence of vasculature leads to defective cells and limits the size of brain organoids. Lack of vasculature also eliminates the endothelial cell signaling from blood vessels, which is needed for proper development of the brain. The vascular cells form a niche for neural progenitor cell development; their absence affects progenitor cell development. Furthermore, efficient delivery of oxygen and nutrients and the removal of metabolites may improve the regionalization of brain organoids, which is mostly missing in current technologies.

**Fig 1:**
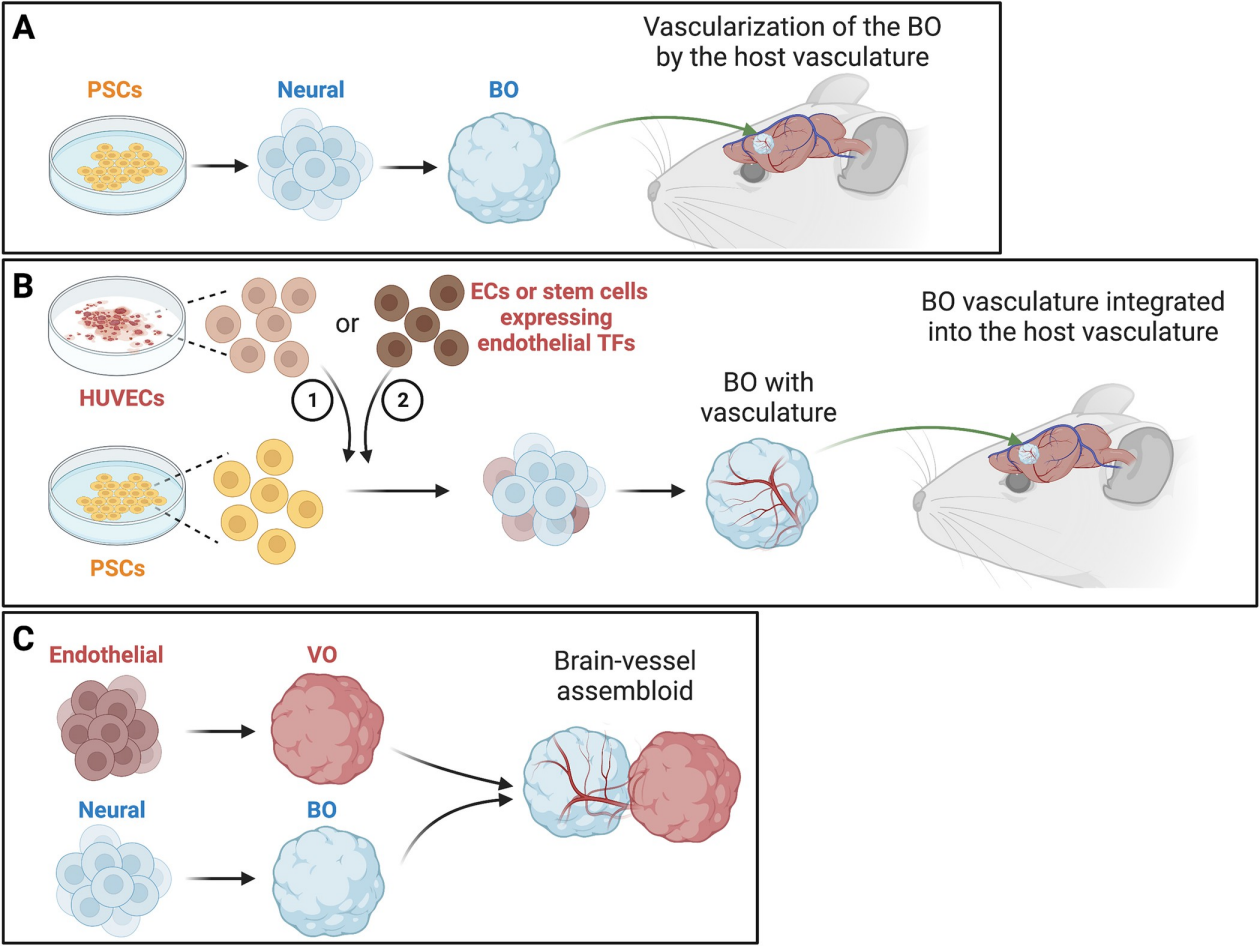
Technologies for vascularizing brain organoids. (A) Transplanting human BOs into the brains of immunodeficient rodents leads to vascularization of the BOs by the host vasculature. BOs are generated from PSCs that are induced to become neural stem cells. (B) Adding vasculature-deriving ECs into developing BOs results in BOs with a vascular system. Neural cells originate from ectoderm, while vascular cells derive from mesoderm. Co-culturing HUVECs with stem cells leads to a vascular system in the BOs. After the HUVEC-BOs are transplanted into the mouse neocortex, the HUVEC-derived vessels integrated into the host vasculature. Adding induced PSC-derived ECs or stem cells expressing endothelial TFs also leads to vessel growth in BOs. (C) Co-culturing human BOs with human blood VO lead to vessel-like structures in the BOs. Graphics are created with BioRender.com. BO, brain organoid; EC, endothelial cell; HUVEC, human umbilical vein endothelial cell; PSC, pluripotent stem cell; TF, transcription factor; VO, vessel organoid.

One approach to vascularizing human brain organoids is to transplant them into the brains of immunodeficient rodents (Fig 1A). This leads to vascularization and improved maturation of the transplanted brain organoids [3,4]. However, transplantation of brain organoids into rodent brains is difficult to scale up. Moreover, gene expression in human vascular cells is not exactly the same as rodent cells, which might cause differences in brain organoid development.

Another approach is to add vasculature-deriving cells into the brain organoids (Fig 1B). Neural and vascular cells originate from ectoderm and mesoderm, respectively. A series of studies have aimed at improving brain organoid technology by integrating non-ectodermal cells into the ectoderm-derived organoids. For example, human brain organoids embedded in Matrigel containing human iPSC-derived endothelial cells grow vessels in them [5]. Expressing endothelial transcription factors in some stem cells promotes neuronal maturation, reduces cell death, and increases the size of brain organoids [6]. Moreover, these induced endothelial cells express tight junction markers and transporters and are surrounded by pericytes and astrocytes, which are characteristics of the blood–brain barrier (BBB).

Taking a different approach to this form of vascularization, Shi and colleagues achieved impressive improvements in brain organoid development in a 2020 *PLOS Biology* study [7]. The authors co-cultured human umbilical vein endothelial cells (HUVECs) with either human iPSCs or embryonic stem cells to make organoid precursors before neural induction, which led to a vascular system formed by the HUVECs in brain organoids (HUVEC-BOs). HUVECs offer 2 benefits. First, they are more accessible than endothelial cells derived from stem cells or most other types of human cells. Second, these cells have a propensity to form tubular structures and are thus commonly used for studying angiogenesis. The HUVEC-BOs had reduced markers for hypoxia and cell death and were larger than non-vascularized brain organoids. Importantly, HUVEC-BOs recapitulated many aspects of neurogenesis and gliogenesis in human cortical development. Vessels first appeared in the region resembling the ventricular zone/subventricular zone, which is similar to the appearance of vasculature in this region before penetrating the cortical plate in human brain development. The HUVEC-BOs had increased functional maturation and synaptic connections and showed an increased expression of P-glycoprotein, an important efflux transporter in the endothelial cells of the BBB. Because P-glycoprotein is not expressed when HUVECs are cultured alone, this result suggests that neural cells induce P-glycoprotein expression in the vascular cells. This finding indicates that the HUVECs not only develop to form vasculature but also might be useful for modeling the BBB.

Since brain organoids vascularized in vitro do not have circulation, Shi and colleagues transplanted the HUVEC-BOs into the mouse neocortex to determine whether the HUVEC-derived vascular system could be integrated into the host vasculature. Endothelial cells derived from HUVECs and the host mouse coexisted in the vessels of the transplanted brain organoids, suggesting that the 2 types of vessels joined to form human–mouse blood vessels in the grafts. Imaging fluorescent dye injected into the vessels of the mice showed steady blood flow in the grafts, demonstrating a functional vasculature in the grafted brain organoids. The extensive testing of the vascularized brain organoids in Shi and colleagues has set a high standard for the field.

Subsequent studies have continued to demonstrate the effectiveness of coculturing human vascular cells and brain organoids to improve brain organoid development. In a 2021 study, coculturing human brain organoids with human blood vessel organoids led to vessel-like structures (Fig 1C) [8]. The endothelial tubes were surrounded by mural cells and astrocytes, resembling the neurovascular tissue in the human cortex. Similar to Shi and colleagues [7], the authors of this study found that the vessel-like structures expressed several markers for tight junctions and astrocytes [8], although they did not observe the morphological characteristics of the BBB. A 2022 study took a similar approach and demonstrated that BBB-like structures formed in brain organoid–vessel organoid (BO–VO) cocultures and even showed selective permeability for a BBB-penetrating peptide [9]. These BO–VO cocultures also contained cells that resembled microglial cells, the immune cells in the brain.

Adding cells from the vascular system has even been demonstrated as an approach for studying pathogenesis. For example, adding stem-cell–derived pericyte-like cells, which contribute to the BBB and express the receptor for SARS-CoV-2, has been used to model SARS-CoV-2 neuropathology [10]. Vascularized brain organoids will be instrumental for studying human diseases that involve brain endothelial cells or pathogenic processes that occur later in life [1].

The progress in developing approaches to vascularizing human brain organoids has been exciting in the past 5 years. The study by Shi and colleagues [7] offers a more accessible and effective vasculature-deriving approach for brain organoids; its full potential has just begun to be explored. Major gaps still need to be filled to achieve the complete vascularization of brain organoids. First, unless they are transplanted into a live animal, current brain organoids lack the pumping mechanisms required for blood flow. Moreover, blood cells are missing from brain organoids in vitro, which not only limits brain organoid development but also precludes studies to determine whether blood or its constituents are essential for brain development. This is especially relevant since the host mice for organoid transplantation are immunodeficient. Second, vascularized brain organoids currently display very limited BBB properties, which will likely affect brain organoid development and restricts their uses in modeling brain disorders. Moreover, current brain organoids have neither cerebrospinal fluid (CSF), which is secreted by the choroid plexus, nor a blood–CSF barrier. Combining vascularized brain organoids and choroid plexus organoids might be an approach to produce a more complete vasculature in brain organoids [11].

We anticipate that these gaps will be narrowed in this booming field. It is likely that disciplines that have not been extensively incorporated, such as biophysics, chemical biology, engineering, and material sciences, will contribute significantly to this endeavor.
